# Evaluation of Terpenes’ Degradation Rates by Rumen Fluid of Adapted and Non-adapted Animals

**DOI:** 10.1007/s13659-020-00289-3

**Published:** 2020-11-23

**Authors:** I. Poulopoulou, I. Hadjigeorgiou

**Affiliations:** 1 Faculty of Science and Technology, Free University of Bolzano, Univeritätsplatz 5, 39100 Bozen – Bolzano, Italy; 2 Department of Nutritional Physiology and Feeding, Faculty of Animal Science and Aquaculture, Agricultural University of Athens, 75 Iera Odos, 11855 Athens, Greece

**Keywords:** Terpenes, Degradation, Rumen, Sheep, Goats

## Abstract

The aim of the present study was to evaluate terpenes degradation rate in the rumen fluid from adapted and non-adapted animals. Four castrated healthy animals, two rams and two bucks, were used. Animals were daily orally dosed for 2 weeks with 1 g of each of the following terpenes, α-pinene, limonene and β-caryophyllene. At the end of each week, rumen fluid (RF) samples were assayed in vitro for their potential to degrade terpenes over time. For each animal, a 10 mL reaction medium (RM) at a ratio 1:9 (v/v) was prepared and a terpenes solution at a concentration of 100 μg/ml each, was added in each RM tube. Tubes were incubated at 39 °C under anaerobic conditions and their contents sampled at 0, 2, 4, 8, 21 and 24 h. RF could degrade terpenes as it was shown by the significantly (P < 0.05) higher overall degradation rates. Individual terpene degradation rates, were significantly (P < 0.05) higher in week 5 for limonene and marginally (P = 0.083) higher also in week 5 for α-pinene. In conclusion, the findings of the present preliminary study suggest that terpenes can be degraded in the rumen fluid.

## Introduction

Terpenes are widely acknowledged for their use as biomarkers that certify the origin and quality of animal products [1–3], while they are also known for their antimicrobial and pharmacological properties [4]. Recently the monoterpenes, limonene [5] and α-pinene [6] and the sesquiterpene β-caryophyllene, which generally accumulated together with monoterpenes, have been evaluated for their ability to, modulate rumen function [7] or even inhibit ruminal digestion [8] and reduce methane emissions from ruminants [9, 10]. However, the efforts to investigate the effect of terpenes on animal physiology and especially on rumen fermentation, are yet not entirely describe the function of terpenes on rumen environment [11]. Recently, a study [12] about the degradation of 17 terpenes and oxygenated monoterpenes, following their addition in the rumen fluid (RF) of dairy goats, showed that degradation rates differed significantly between terpenes. The authors attributed the observed differences to the structure of the molecule and more specifically to the presence of an oxygen ring. Additionally [13], demonstrated that anaerobic degradation of monoterpenes like α-pinene and limonene was possible with the use of *Pseudomonas citronellolis* cultivated in anaerobic conditions, while others [14], reported that denitrifying and fermenting bacteria affected anaerobic degradation of terpenes. Additionally, when in an in vitro study the ability of various individual essential oils (EO), some of which are the EO of oregano, rosemary, mind, cinnamon, dill, eucalyptus and their combinations to alter rumen microbiota was tested, authors [10] found that rumen population had different sensitivity to different EO combinations and is dose dependent. Additionally, they also concluded that the effect may not be attributed necessarily to the major EO components (e.g. monoterpenes), but to the EO containing a phenolic or carbonyl group. For example although eugenol can be found in cinnamon EO, its high biological activity attributed to the presence of carbonyl group which determined in the major component of cinnamon EO the phenylpropanoid *trans*-cinnamaldehyde [10]. On the contrary, researchers [15] who studied in vitro the effect of a mixture of EO (oregano, cinnamon, thyme, orange peel) on rumen fermentation, found no effect on microbial population and partly attributed the result on the short experimental length (24 h). They also concluded that both, the type of EO mixture used and the adaptation period of rumen bacteria to EO mixture, might have an important role on modifying rumen microbial population if tests performed for a longer time length. That conclusion also supported by a study [9] that refers that the longer the exposure of microbial population to EO, the higher the possibilities of the microbial population to alter its synthesis and finally to adapt to the added compounds with time [16].

However, the research that tests the effect of a mixture of EO on rumen microbial fermentation is mostly focused on cattle. Additionally, the effect of individual terpenes degradation rate (e.g. monoterpenes and/or sesquiterpenes) by small ruminants (sheep and goats) is still not fully described, although there have been studies that tried to indirectly determine the effect of terpenes on the rumen microflora [17–19]. Thus, for the present study, α-pinene, limonene and β-caryophyllene, which are terpenes that have a different molecular structure and are representative in South-eastern Mediterranean flora were selected. The tested hypothesis was to assess in vitro the potential of rumen fluid (RF) originated from, (a) non-adapted, to a terpenes diet mixture, (b) adapted, during a 2-week adaptation period to an orally administered terpene mixture, donors animals to degrade the terpenes.

## Results

The chemical analysis of the diet supplied to the animals on a dry matter (DM) basis is shown in Table 1. Since no differences in degradation rates between rams and bucks were observed, data were pooled over animal species and experimental weeks. The results showed that mean degradation rates for each terpene differed significantly (P < 0.05) between the rumen fluid (RM-RF) and no rumen fluid (RM-PBS) samples (Table 2). In particular, rumen fluid addition (RM-RF test group) resulted in significantly higher terpene degradation rates compared to the control test group with no RF addition (RM-PBS).

**Table 1:** Chemical composition of animal’s diet at DM basis

Feedstuff	DM (%)	Fat (%)	Protein (%)	Fiber (%)	Ash (%)
Alfalfa hay	90.1	1.7	20.3	29.2	9.1
Wheat straw	88	1.4	2.5	39.6	5.4
Concentrate mixture	88.5	3.1	11.9	10.5	6.4

**Table 2:** Overall pooled mean terpene degradation rates (h^−1^) (mean ± SE) from pooled data over animal species and experimental weeks for rumen fluid (RM-RF) and control (RM-PBS) samples

Substance	Test group	Mean degradation rate (h^−1^)	P-value*
α-Pinene	RM-RF	0.036 ± 0.0067	0.013
	RM-PBS	0.001 ± 0.0000	
Limonene	RM-RF	0.05 ± 0.0083	0.019
	RM-PBS	0.001 ± 0.0002	
β-Caryophyllene	RM-RF	0.043 ± 0.0107	0.034
	RM-PBS	0.001 ± 0.0002	

*P < 0.05

**Fig. 1:**
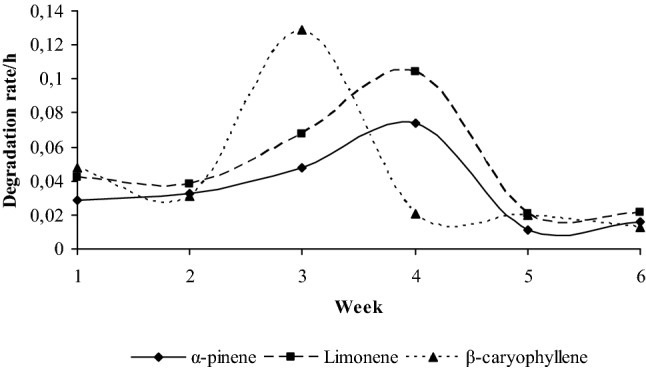
The evolution of pooled mean terpenes degradation rates (h^−1^) by rumen fluid samples during the 6 weeks of the experiment

Mean degradation rates of the individual terpenes showed a different pattern between weeks (Fig. 1). Degradation of α-pinene across weeks showed an increasing pattern until week 4 and the same was observed for limonene, though at a higher degradation rate. On 5th week, degradation rates of the above mentioned terpenes decreased and remained at the same level until the last week of the trial. However, β-caryophyllene degradation rate increased in the 3rd experimental week, rapidly decreased on the 4th week of the experiment and remained constant until the end of the trial.

For all animals, the comparison of degradation rates followed similar patterns between the experimental weeks. Terpenes pooled mean degradation rate of α-pinene in rumen fluid is presented in Table 3. Between the experimental weeks, there were no significant differences. However, there was a trend for a higher degradation rate (P = 0.084) in the 5th week, which was the first week after the end of terpenes oral administration and week 2, where terpenes had not been yet administered to the animals. In addition, a trend for higher α-pinene degradation rate (P = 0.091) was also noted in week 3 where terpene administration started and the 5th week where, there was no terpene dosing.

**Table 3:** Comparison between weeks of pooled mean α-pinene degradation rates (h^−1^) (mean ± SE) from data pooled over animal species for rumen fluid samples during the experiment

Week	2	3	4	5	6
1	− 0.004 ± 0.0048	− 0.019 ± 0.0086	− 0.046 ± 0.0244	− 0.018 ± 0.0057	0.013 ± 0.1451
P-value	0.983	0.507	0.657	0.257	0.987
2		− 0.015 ± 0.0085	− 0.042 ± 0.0244	− 0.022 ± 0.0055	0.017 ± 0.0144
P-value		0.715	0.730	0.084	0.932
3			− 0.027 ± 0.0254	− 0.037 ± 0.0091	0.032 ± 0.0161
P-value			0.965	0.091	0.598
4				− 0.063 ± 0.0246	0.585 ± 0.280
P-value				0.401	0.539
5					− 0.005 ± 0.0148
P-value					1.000

Degradation rate of limonene (Table 4) was significantly (P < 0.05) higher in week 5 compared to the 1st experimental week. In addition, a trend (P = 0.064) for higher limonene degradation rate was also noticed between the 3rd week which was the first week of terpenes oral administration and 5th week of the experiment. Degradation rate of β-caryophyllene was not significantly notified during the 6 weeks of the experiment (Table 5).

**Table 4:** Comparison between weeks of pooled mean limonene degradation rates (h^−1^) (mean ± SE) from pooled data over animal species for rumen fluid samples during the experiment

Week	2	3	4	5	6
1	0.004 ± 0.0084	− 0.026 ± 0.0087	− 0.062 ± 0.0273	0.021 ± 0.0028*	0.021 ± 0.0093
P-value	1.000	0.317	0.505	< 0.05	0.528
2		− 0.029 ± 0.0122	− 0.066 ± 0.0286	0.018 ± 0.0090	0.170 ± 0.1264
P-value		0.398	0.481	0.619	0.897
3			− 0.036 ± 0.0287	0.047 ± 0.0092	0.046 ± 0.0128
P-value			0.909	0.064	0.102
4				0.083 ± 0.0275	0.083 ± 0.0289
P-value				0.297	0.304
5					0.001 ± 0.0097
P-value					1.000

*P < 0.05

**Table 5:** Comparison between weeks of pooled mean β-caryophyllene degradation rates (h^−1^) (mean ± SE) from pooled data over animal species for rumen fluid samples during the experiment

Week	2	3	4	5	6
1	− 0.017 ± 0.0080	− 0.022 ± 0.0313	− 0.027 ± 0.0052	− 0.027 ± 0.0133	0.035 ± 0.0093
P-value	0.557	0.392	0.182	0.597	0.145
2		− 0.099 ± 0.0317	− 0.009 ± 0.0072	− 0.011 ± 0.0142	0.018 ± 0.0106
P-value		0.268	0.880	0.998	0.732
3			− 0.011 ± 0.0311	− 0.011 ± 0.0334	0.117 ± 0.0320
P-value			0.218	0.209	0.178
4				− 0.001 ± 0.0128	0.008 ± 0.0086
P-value				1.000	0.980
5					0.008 ± 0.0150
P-value					1.000

## Discussion and Conclusion

Until recently, studies focused on terpenes, dealt mainly with their antimicrobial properties [20], their use as biomarkers in identification of animal products [21] or their influence on animal behaviour [22]. Additionally, although the well reported differences in diet selection [23], intake and nutritional physiology between cattle and small ruminants [24], research data regarding terpenes fate on sheep and goats are limited [25, 26]. In particular, their degradation by rumen microorganisms and their effect on rumen microflora is mainly focuses on cattle. The research findings that determined different composition of rumen microorganisms when the same diet was fed in cows and goat or sheep [27, 28], verify that there are species differences in the metabolism of nutrients in the rumen. However, it seems that terpenes also affect this procedure indicating that, those substances should be further investigated.

Since animal’s welfare is of key importance, the methods followed in the present experiment had the less possible intervention on experimental animals and therefore in vitro experimentation was the procedure of choice. The anaerobic system used in the present experiment followed the principles of a traditional non dynamic in vitro system that has been proposed by other researchers [22]. Moreover, the addition of a terpenes mixture to the animals’ diet and the use of rumen fluid for further trials come to a step closer to the in vivo situation, by using a medium that contains concentrations of the tested substances that are realistic. Other authors [9] also supported that the results from in vitro experiments can investigate more precisely the effect of phytochemicals in batch or continuous cultures since their distribution is uniform and the microorganisms directly exposed to the tested substance.

The results of the present in vitro experiment, reveal that terpenes degradation rates were affected by the origin of RF from adapted as well as from non-adapted to terpenes donor animals. The concentrations of terpenes mixture (100 μg/ml) used in the present trial were in line with previously published relevant studies [25]. However, what needs to be noted is that in most of the published data a mixture of EO, originated from plant extraction, was used while in the present experiment the effect of pure substances on rumen environment was investigated. Moreover, when a mixture of EO at a concentration of less than 100 ppm was added on pure cultures of rumen microorganisms it was found that it inhibits the growth of most cultures of ruminal bacteria and ruminal fungus while ruminal protozoa remained unaffected a fact that might be attributed to specific differences between cattle and sheep [29]. Additionally, the results of the present study, where rumen fluid (RM-RF) resulted in significantly higher terpene degradation rates compared to the control (RF-PBS), could also be partly attributed to the ability of terpenes to manipulate rumen environment. The very low degradation rates, observed in the RF-PBS samples, are considered as the effects of uncontrolled experimental errors, thus indicating a satisfactory estimation of terpenes’ degradation rates in rumen fluid.

The statistically significant differences observed in degradation rates of terpenes in RF, can also be attributed to the possible adaptation of rumen environment to the tested substances. That has also been reported in similar studies where the high degradation rates of terpenes in rumen environment attributed to the adaptation of rumen microorganisms to the new substance [11, 12]. Additionally, what needs to be stressed is that fiber adapted microflora had a faster potential to degrade terpenes reaching to a plateau in the first 3 h after terpene mixture administration, while starch adapted microorganisms reached a plateaus 6 h after administration. However, the complex procedure that affects terpenes degradation in the rumen and the biotic and environmental variables can also influence that procedure.

The statistical significant degradation rates of limonene (P < 0.05) in RF, in the present experiment agree with the results from other researchers [30], who tested in vitro increasing concentrations of a mixture of EO (0, 5, 50, 500, 5000 mg/l) consisted of thymol, eugenol, limonene, guaiacol and vanillin, in cow’s rumen fluid and observed minor alterations in degradation rates even at the highest inclusion level [31]. In contrast, different are the findings of those [32] who screened the effect of plant extracts (anise oil, cade oil, capsicum oil, cinnamon oil, clove bud oil, dill oil, fenugreek, garlic oil, ginger oil, oregano oil, tea tree oil, and yucca) on dairy cattle microbial fermentation in a continuous culture system, in lower concentrations (0, 3, 30, 300, 3000 mg/l). They found that terpenes had the ability in high doses (300, 3000 mg/l) to affect rumen microbial environment and in some cases to cause even detrimental effects on rumen microorganism. The trend determined for α-pinene degradation rate between weeks 2 and 5, agree with limited effects in terpene degradation rates reported in other studies [16, 30], where the lack of effect on fermentation attributed either to low inclusion level used or to the lack of impact of the investigated compound to the rumen ecosystem.

Furthermore, it has been reported [24] that when α-pinene and limonene and other mono and susquiterpenes like sabinene, camphene, terpinolene, linalool, myrcene key components of scrub species (*Artemisia tridentata*, *Flourensia cernua*, *Juniperus* species) added in a mixture of EO in rumen fluid at a concentration of 10 mg/ml and analysed immediately, their recovery rates determined at a concentration of 50% of the initial one. For β-caryophyllene recovery rate was determined at about 30% of the initial dose. These findings are in agreement with the results of those [23] who measured similar recovery rates for the above two terpenes (limonene and β-caryophyllene) after 24 h incubation in goats’ rumen fluid and determined high capacity of rumen microbial ecosystem to degrade α-pinene. An incubation period of 24 h like the one applied in this study is in line with the time period used among research studies that investigate terpene degradation in rumen environment [33, 34]. However, the differences observed between the degradation rates of above mentioned studies and the results of the present study can be attributed to the different concentration of terpenes used. That was also stated by those [35], who showed that the effect of terpenes in nitrogen metabolism of rumen microorganisms is dose depended and suggested that further research is required to determine the dose and the time of adaptation to EO required to observe alteration in rumen metabolism.

In conclusion, the findings of the present small scale study suggest that terpenes can be degraded by rumen fluid both in adapted and non-adapted animals. However, it appears that the observed differences in terpenes degradation rates depend on the individual terpene investigated and the in vivo rumen fluid sampling time span. In this sense, the fact that from the three terpenes investigated in the present study, there were no significant effects in the degradation rates of a-pinene and β-caryophyllene between the experimental weeks, could be attributed to the adaptation of rumen microflora, within the weeks of terpene oral administration to the animals.

## Experimental Section

### Animals, Diets and Design

The trial was conducted at the experimental facilities of the Department of Nutritional Physiology and Feeding, of the Agricultural University Athens. Four adult healthy male animals—two rams (BW 56 kg) and two bucks (BW 65 kg) were used as rumen fluid (RF) donor animals for the study. The animals were all housed in the same outdoors pen with an open shade and fed in group. The ration that fed to the animals twice daily at 09:00 h and 16:00 h, was based on alfalfa hay, wheat straw and a commercial pelleted concentrate mixture which consisted, on a dry matter basis, of: 10% crude protein; 4% fiber; 1.2% Ca; 0.9% P, vitamins and minerals. The diet was calculated to cover the animals’ requirements for maintenance. Animals had free access to fresh tap water during the whole experiment and treated humanely according to the ethics guidelines of the Agricultural University of Athens.

**Fig. 2:**
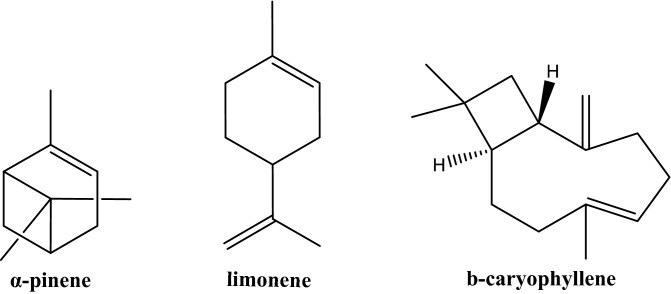
Chemical structures of α-pinene, limonene and β- caryophyllene

The experiment lasted 6 weeks. During weeks 1 and 2 the animals were receiving the diet without administration of terpenes. On weeks 3 and 4 bucks and rams were orally dosed each morning with 1 g of each of the following terpenes: α-pinene, limonene, β-caryophyllene (Sigma Aldrich: No.2320878 USA, No.2278135 Switzerland, No.2017461 Spain, respectively) (Fig. 2). An appropriate 10 ml mixture of the three terpenes in vegetable oil (soybean oil) was prepared to provide 1 g of each of the above mentioned terpenes. Similarly with weeks 1 and 2, during the last two experimental weeks (weeks 5 and 6), animals were receiving the diet without the administration of terpenes. In order to follow the same protocol throughout the duration of the trial and avoid aversive behaviour from the animals, on weeks 1, 2 and 5, 6, 10 ml of vegetable oil without the terpenes mixture was also orally administered to the animals.

At the end of each week, samples of rumen fluid were taken from each of the four animals with the use of an oesophageal catheter under light sedation. The oesophageal catheter method was chosen as the most appropriate method for rumen fluid sampling, taking into consideration main animal welfare principles. The samples were collected in sterile bottles and transferred immediately to the laboratory where rumen solids were filtered using double layer cheese cloth and RF was collected. Subsequently, a 10 ml reaction medium (RM) for each RF sample was made as follows: 9 ml of RF were placed in a PTFE capped culture tube followed by addition of 1 ml PBS-Tween 20 solution containing 100 μg/ml of each of the following terpenes: α-pinene, limonene and β-caryophyllene. Additionally, appropriate controls were made by using 9 ml of phosphate buffered saline (PBS) instead of RF and 1 ml of PBS-Tween 20 containing 100 μg/ml of each of the following terpenes: α-pinene, limonene and β-caryophyllene. Therefore, RM containing RF and RM containing PBS will be denoted here as RM-RF, and RM-PBS respectively. Subsequently, all RM tubes were placed, in an incubator under anaerobic conditions (10% CO_2_ and 90% N_2_) set at 39 °C for 24 h. One ml subsamples were taken at 0, 2, 4, 8, 21 and 24 h after terpenes addition from each RM tube and placed in PTFE caped vials. Samples were stored at − 80 °C until analysis for terpenes concentration.

### Rumen Fluid Analysis

RM samples were extracted using a dichloromethane/methanol 2:1 solution, according to the technique reported by [23] and modified by us, as follows: in 1 ml of RM, placed in a PTFE capped vial, 500 μl of the dichloromethane/methanol solution were added. After centrifugation (2000×*g* for 7 min) the liquid phase was collected. The residue was extracted twice with 500 μl of dichloromethane/methanol and once with 250 μl of the same solution. The three extracts were combined and 1 ml was transferred in auto sampler vials. All samples were analyzed by GC–MS in duplicate.

A Hewlett-Packard 5890 gas chromatograph, equipped with a splitless injector and a HP 5 capillary column (25 m × 0.2 mm I.D.) coated with a 5% phenylmethyl silicone phase (0.33 μm), was used. The chromatograph was coupled with a mass spectrometer (MS) (MSD HP 5970) operating under electron ionization (EI). The samples were injected through an automatic auto-sampler (ALS 7673) and the whole analytical procedure was controlled with the program MS 3.2 of Pascal Chemstation (HP 59970).

The target compounds were identified on the chromatograph as follows: oven temperature was programmed up to 280 °C. To elute the higher molecular mass, compounds were extracted from the matrix using the following temperature program: 35 °C for 1 min; increase by 15 °C/min up to 75 °C and hold constant for 1 min; increase 3 °C/min up to 90 °C; increase 20 °C/min up to 180 °C; increase 25 °C/min up to 280 °C and hold constant for 5 min. Helium was used as the carrier gas with a flow rate of 1 ml/min.

### Statistical Analysis

For each one of the three terpenes the respective residual concentrations determined in the RM for the sampling time points were used for the calculation of the terpene degradation rate in each experimental week. The following equation appeared to fit the data reasonably well:

$${\text{y}}_{{\text{t}}} = {\text{a}}*{\text{exp}}^{{{\text{bt}}}}$$

where b is terpenes degradation rate, t is the reaction time (h), a is the initial concentration of each terpene in RM at time 0 h, y is the respective terpene concentration at a time point in question. For each experimental week, mean degradation rate was estimated using the results of the different sampling time points (0, 2, 4, 8, 21, 24 h) for each one of the three terpenes.

Terpenes degradation rates of RM-RF and RM-PBS samples were compared using non-parametric (Mann–Whitney) t-test. Comparisons between experimental weeks were performed using one-way analysis of variance. Individual differences were tested post–hoc using Bonferroni test, since Levene’s test indicated equality of variance. All statistical analyses were conducted using the Statgraphics (version 2.7) software [36].
